# Derivation of a Simple Risk Scoring Scheme for Prediction of Severe Dengue Infection in Adult Patients in Thailand

**DOI:** 10.3390/medsci13040244

**Published:** 2025-10-26

**Authors:** Surangrat Pongpan, Patcharin Khamnuan, Pantitcha Thanatrakolsri, Supa Vittaporn, Punnaphat Daraswang

**Affiliations:** 1 Faculty of Public Health, Thammasat University, Lampang 52190, Thailand; surangrat.p@fph.tu.ac.th (S.P.); pantitcha.o@fph.tu.ac.th (P.T.); supa.v@fph.tu.ac.th (S.V.); 2 Thammasat University Research Unit in Environment, Health and Epidemiology, Lampang 52190, Thailand; 3 Buriram Hospital, Muang, Buriram 31000, Thailand; punnapath.da@cpird.in.th

**Keywords:** risk score, clinical prediction rule, severe dengue, predictive model, adult patients, Thailand

## Abstract

Background/Objectives: Severe dengue infection remains a major public health burden in Thailand, where timely identification of high-risk patients is essential for effective clinical management. Existing predictive models are often complex and less feasible in routine practice. This study aimed to develop a simple risk scoring system to predict dengue severity based on patient characteristics and routine clinical data. Methods: Retrospective data of adult dengue patients from nine general hospitals in Thailand from 2019 to 2022 were reviewed. Dengue infection was classified into two groups using the WHO 2009 modified criteria: non-severe (n = 577) and severe (n = 107). Demographic data, clinical characteristics, and laboratory findings were analyzed using logistic regression. Regression coefficients of significant predictors of severe dengue were converted into weighted item scores. Total scores were categorized into three risk levels based on probability distribution cut-off points. Results: The severity score stratified patients into three risk groups with significantly different prognoses: ≤2.0 points (low risk), 2.5–5.0 points (moderate risk), and ≥5.5 points (high risk). The positive likelihood ratios for low-, moderate-, and high-risk groups were 0.12, 1.05, and 28.76, respectively. The distribution of severity scores differed significantly between non-severe and severe cases. The scoring system discriminated between non-severe and severe dengue with an area under the receiver operating characteristic curve (AUROC) of 88.04% (95% CI, 83.99–92.08). Conclusions: The derived dengue severity scoring system classified patients into low, moderate, and high risk with excellent discriminatory performance, effectively distinguishing non-severe from severe dengue infection.

## 1. Introduction

Dengue is a mosquito-borne viral disease caused by four distinct serotypes of the dengue virus (DENV 1–4), transmitted primarily by *Aedes* mosquitoes. Since immunity is serotype-specific, individuals may experience multiple infections throughout their lifetime because dengue virus comprises four distinct serotypes (DENV-1 to DENV-4), and infection with one does not provide lasting immunity against the others [1]. The global incidence of dengue has increased substantially over recent decades, affecting more than 100 countries and causing tens of thousands of deaths each year, making it one of the fastest-growing mosquito-borne diseases worldwide [2].

Southeast Asia, including Thailand, bears a disproportionately high disease burden, with outbreaks occurring predominantly during the rainy season. Environmental factors, urbanization, and climate change further amplify dengue transmission in this region [3]. In 2024, Thailand reported 105,206 dengue cases with 90 associated deaths, reflecting a case fatality rate of 0.09% [4]. Although this mortality rate appears low, the large number of infection results in considerable health and economic impacts [5]. In early 2025, reported cases rose sharply, underscoring Thailand’s hyperendemic status with multiple circulating serotypes [6].

Clinically, approximately 25% of dengue infections are symptomatic, and around 5% of these progress to severe disease, which can be fatal. The nonspecific nature of early symptoms complicates timely diagnosis; however, early recognition of warning signs and prompt fluid management can reduce mortality to below 0.5% [7]. Therefore, reliable clinical tools for early risk stratification are crucial in preventing disease progression.

In response to this need, several clinical scoring systems have been developed globally to predict severe dengue outcome. For example, Lee IK et al. proposed a simple clinical score in Taiwan [8]. Marois et al. developed a bedside score in New Caledonia [9], and Bhandari S et al. designed the SMS Dengue Severity Score in India [10]. Other models have been proposed in Indonesia [11], Singapore [12], and Vietnam [13], each demonstrating promising predictive performance within their respective settings.

Despite these advances, many existing models are complex, rely on extensive laboratory parameters, or were developed in populations with epidemiological characteristic different from those of Thai patients. These limitations restrict their generalizability and practical use in local healthcare settings. Hence, a simple, validated, and locally applicable scoring system is needed to improve risk assessment among Thai adults with dengue.

Our previous multicenter studies in Thailand identified key predictors of severe dengue—namely, age < 55 years, severe bleeding, pleural effusion, and platelet count ≤ 100,000/µL [14]. Building on these findings, the present study aimed to develop a practical and easy-to-use clinical scoring algorithm for adult dengue patients in Thailand. This scoring system, derived from routinely available clinical and laboratory parameters, classifies patients into low, moderate, and high-risk categories for severe dengue. By enabling early risk assessment, it supports timely clinical decision-making, optimal resource allocation, and improved patient outcomes.

## 2. Materials and Methods

### 2.1. Study Design and Participants

This study employed a retrospective case–control design to develop a clinical prediction rule. Medical records of adult patients (aged ≥ 15 years) with laboratory-confirmed dengue infection were reviewed from nine hospitals across Thailand between 2019 and 2022, including Tak Hospital, Nakhon Pathom Hospital, Phra Nakhon Si Ayutthaya Hospital, Rayong Hospital, Sisaket Hospital, Surin Hospital, Loei Hospital, Trang Hospital, and Phatthalung Hospital. Dengue infection was confirmed by a positive NS1 antigen, RT-PCR, or dengue IgM/IgG antibody test. Patients were identified using the following ICD-10 codes: A90 (Dengue fever, DF), A91 (Dengue hemorrhagic fever, DHF), A910 (Dengue hemorrhagic fever with shock, DSS), A97.0 (Dengue without warning signs), A97.1 (Dengue with warning signs), and A97.2 (Severe dengue).

Patients who met the criteria for severe dengue were classified as the case group (n = 107), while those with non-severe dengue (n = 577) were selected as the control group. The classification was based on disease severity rather than an exhaustive inclusion of all dengue cases during the study period. Disease severity was categorized according to the 2009 WHO dengue classification guidelines [15]. Severe dengue was defined by either severe plasma leakage, resulting in shock and/or respiratory distress, or by severe organ impairment such as pulmonary edema, disseminated intravascular coagulation, encephalopathy, liver failure, and/or renal failure. Patients without any of these severe manifestations were categorized as non-severe. This design allowed for a retrospective comparison of clinical and laboratory characteristics between severe and non-severe cases.

In this study, “adults” were defined as individuals aged ≥ 15 years, following the national epidemiological and clinical practice standards in Thailand, where persons aged 15 years and older are generally classified as adults in public health and hospital-based systems.

### 2.2. Data Collection Process

Medical files of adult patients with dengue infection were retrieved from the hospital database. All clinical and laboratory variables were collected at the time of hospital admission, before the final classification of severe dengue was made.

Data collection included the following:

(1) Demographic data: gender, age, body mass index (BMI), and underlying diseases (including hypertension, diabetes mellitus, dyslipidemia, chronic liver disease, chronic kidney disease, and other physician-diagnosed chronic conditions).
(2) Clinical characteristics: fever, headache, myalgia, Retro-orbital pain, bone pain, Joint pain, abdominal pain, vomiting, cough, diarrhea, petechiae, rash, epistaxis, bleeding per gums, severe bleeding, hepatomegaly, pleural effusion, rapid weak pulse, systolic blood pressure, diastolic blood pressure, pulse pressure.
(3) Laboratory findings: hemoglobin, hematocrit, white blood cells, platelets, neutrophils, lymphocytes, aspartate aminotransferase (AST), alanine aminotransferase (ALT).

### 2.3. Study Size Estimation

The sample size was calculated using 10 outcome events per predictor variable (the EPV method) [16]. Based on the other scoring systems, [14] which collectively draw upon 32 variables, a total of 320 patients were needed.

### 2.4. Data Analysis

In developing the prediction model, all potential clinical and laboratory variables were initially analyzed using univariable logistic regression to determine their association with severe dengue infection. Variables with a *p*-value of <0.05 were subsequently entered into a multivariable logistic regression model to identify independent predictors of severe dengue. The regression coefficients obtained from the multivariable model were then transformed into item scores by dividing each coefficient by the smallest coefficient in the model and rounding to the nearest integer, following established clinical score development methods [17,18].

A receiver operating characteristic (ROC) curve was constructed to evaluate the discriminative ability of the derived prediction model, and the Hosmer–Lemeshow chi-square goodness-of-fit test was performed to assess model calibration [19]. Based on the predicted probability distribution, cut-off scores were determined to classify patients into three severity groups: low, moderate, and high risk of severe dengue infection. The diagnostic performance of each category was then evaluated by calculating sensitivity, specificity, positive predictive value (PPV), negative predictive value (NPV), positive likelihood ratio (LR+), and negative likelihood ratio (LR−) [20,21].

Exact probability tests and independent *t*-tests were used to compare baseline characteristics. Severity scores were assigned for all patients in the development dataset. The area under the receiver operating characteristic curve (AuROC) was calculated to assess the performance of the scoring system in the development cohort. Probability curves for each severity level were plotted to demonstrate the discriminative ability of the model. Results were expressed as adjusted odds ratios with 95% confidence intervals (CIs). All statistical tests were two-tailed, and a *p*-value of <0.05 was considered statistically significant. All analyses were performed using Stata version 12.1 (Stata Corp, College Station, TX, USA).

Although the variable “age” did not reach statistical significance in the univariable analysis, it was retained in the final model based on its established clinical relevance and supporting evidence from previous studies demonstrating an association between older age and increased disease severity in dengue infection. The inclusion of age aimed to ensure that the model captured clinically meaningful predictors rather than relying solely on statistical criteria.

In the development of the predictive model, only the main effects of individual explanatory variables were considered. Potential interactions between variables were not formally tested in order to maintain model simplicity and ensure ease of application in routine clinical practice. Future studies may explore possible interaction effects using larger datasets or alternative modeling approaches.

### 2.5. Missing Data Management

As this study utilized retrospective medical records from nine hospitals, some variables had incomplete data. To ensure data quality, variables with more than 20% missing values were excluded from further analysis. For variables with less than 20% missingness, complete-case analysis was applied. Missingness was assessed and found to be random with no significant association with disease severity. Therefore, no imputation methods were performed. This approach ensured that the predictive model was developed based on reliable and consistently available data across study sites.

## 3. Results

### 3.1. Patient Characteristics

Based on the defined classification criteria, patients were divided into two groups: severe (n = 107) and non-severe (n = 577). Significant differences were observed between the two groups in several clinical and laboratory parameters, including headache, abdominal pain, bleeding episodes, pleural effusion, rapid or weak pulse, systolic and diastolic blood pressure, pulse pressure, platelet count, aspartate aminotransferase (AST), and alanine aminotransferase (ALT) levels (Table 1).

### 3.2. Significant Predictors

We previously used multivariable logistic regression to derive prognostic factors for severe dengue in a retrospective collected cohort of 684 adult dengue patients hospitalized. The four independent predictors identified in this study were age < 55 years (OR = 6.13, *p* = 0.054), severe bleeding (bleeding from gastrointestinal tract, hematemesis, melena, menorrhagia, hematuria) (OR = 20.75, *p* < 0.001), Pleural effusion (OR = 10.23, *p* < 0.001), and Platelet ≤ 100,000 (/µL) (OR = 3.62, *p* = 0.035) (Table 2).

Although age did not strictly meet the predefined statistical criterion, it was retained in the final model due to its recognized clinical relevance in predicting disease severity in adult dengue patients.

### 3.3. The Scoring System Development

A score was calculated for each patient by adding the points corresponding to predictors. The item scores range from 0 to 2.5 points, yielding a total score ranging from 0–7 (Table 3).

### 3.4. Clinical Predictions

By classifying the severity scores into 3 risk categories with significantly different prognoses: scores ≤ 2.0 points (low risk), scores 2.5–5.0 points (moderate risk), and scores ≥ 5.5 (high risk) with the likelihood ratio of positive (LHR+) 0.12, 1.05, 28.76, respectively. The mean total score of the severe group and the non-severe group were 4.6 ± 1.5 and 2.5 ± 1.2 (*p* < 0.001) (Table 4).

To further evaluate the diagnostic performance and clinical applicability of the dengue severity risk score, additional performance metrics were calculated at different score thresholds. The lower cut-off point (≥2.5 points), the model achieved high sensitivity (93.5%) but moderate specificity (52.3%), with a positive predictive value (PPV) of 26.7% and negative predictive value (NPV) of 97.7%. At the higher cut-off (≥5.5 points), sensitivity decreased (44.9%) but specificity markedly improved (98.4%), with corresponding PPV and NPV of 84.2% and 90.6%, respectively. These results indicate that the lower threshold is useful for early screening (rule-out severe dengue), whereas the higher threshold is better suited for confirming severe dengue and guiding hospital admission decisions (Table 5).

### 3.5. Discrimination

**Figure 1:**
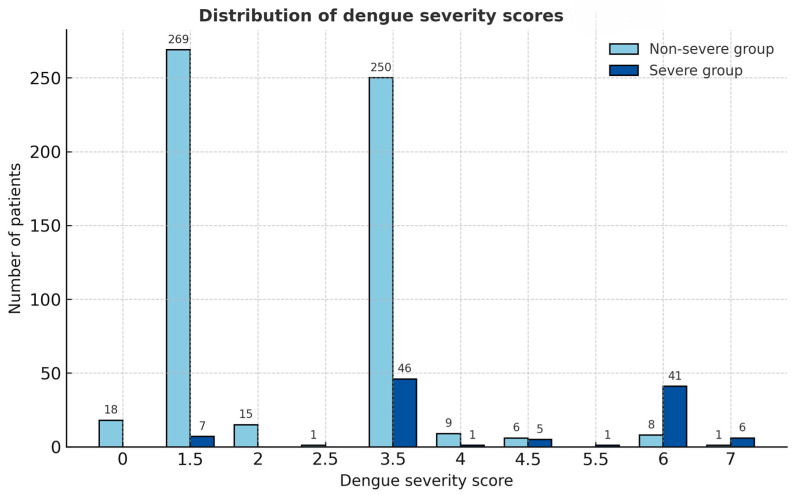
Distribution of dengue severity scores among severe and non-severe groups. Bars represent the number of patients in each score category, illustrating the clear separation between the two groups based on the derived dengue severity score.

**Figure 2:**
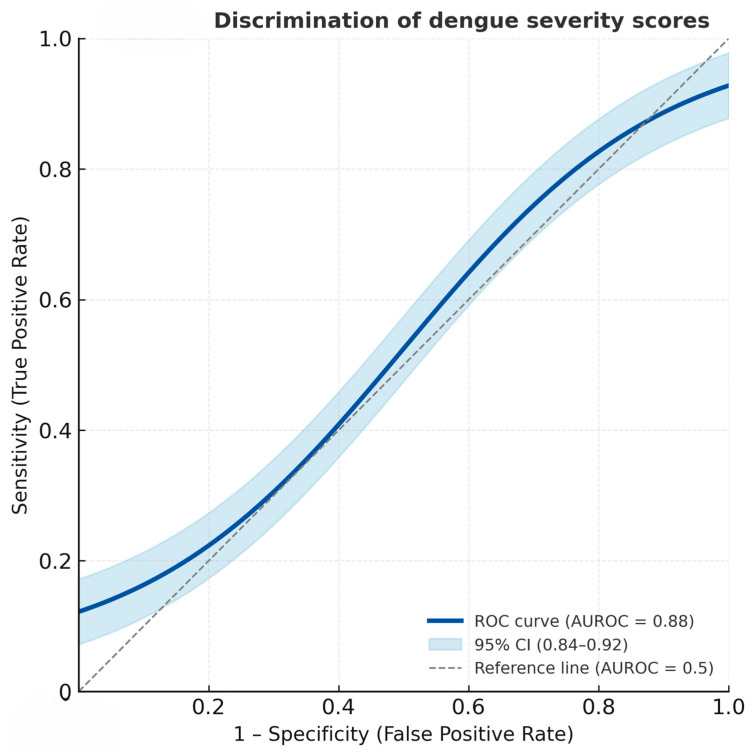
Receiver operating characteristic (ROC) curve showing the discriminatory performance of the dengue severity score for predicting severe dengue. The solid line represents the ROC curve, and the shaded area denotes the 95% confidence interval (AUROC = 0.88, 95% CI: 0.84–0.92).

The derived severity score distribution differed clearly between the two severity groups (Figure 1). Patients with severe dengue had notably higher total scores, clustering predominantly in the higher score categories (≥3.5), whereas non-severe cases were mostly distributed in the lower score range (≤2.0). This visual distinction supports the score’s ability to discriminate between severe and non-severe dengue, consistent with the high area under the receiver operation curve (AUROC) of 88.04% (95% CI = 83.99, 92.08) (Figure 2).

## 4. Discussion

This study developed a simple practical scoring system for predicting severe dengue in adult patients in Thailand. The score was derived from four routinely available parameters—age < 55 years, severe bleeding, pleural effusion, and thrombocytopenia—allowing for easy implementation in resource-limited settings.

In the present study, age was retained as an explanatory variable despite not fully meeting the statistical inclusion threshold, as younger age has been reported in previous studies to be associated with more severe forms of dengue infection among adults. This decision was made to ensure that clinically meaningful factors were not excluded from the model.

The principal strength of the proposed scoring system lies in its simplicity and applicability in real-world clinical settings. Unlike more complex models that require advanced laboratory markers or imaging studies, this score is based solely on parameters that are routinely assessed in all dengue patients. These can be readily obtained at the point of care without specialized investigations, making the score particularly well-suited for use in community and provincial hospitals where advanced diagnostic resources are often limited.

In practice, the score provides a clear framework for clinical decision-making by effectively stratifying patients into three risk subgroups. The selected cutoff points for these classifications are as follows:

1. Low-risk group (score ≤ 2.0 points): This group represents the mildest form of the disease. Patients in this category are suitable for outpatient management or can be discharged with advice for close self-monitoring of any abnormal signs or symptoms, and scheduled for a follow-up appointment.
2. Moderate-risk group (score 2.5–5.0 points): Patients in this subgroup require a higher level of care. They should be referred to a hospital or admitted for in-patient medical treatment and monitoring. However, the “moderate risk” category has limited discriminative power, and should be interpreted with clinical judgment.
3. High-risk group (score ≥ 5.5 points): This classification identifies patients at high risk of developing severe dengue. These individuals require immediate hospitalization for close observation and may need intensive emergency treatment to manage potential complications.

Beyond individual patient care, the scoring system has broader implications for the healthcare system. Seasonal dengue epidemics in Thailand often overwhelm hospital capacity. By distinguishing patients who can be safely treated as outpatients from those who require hospitalization, the score supports rational allocation of limited resources. In rural and provincial hospitals, this prioritization may help avoid overcrowding and ensure that critical resources reach the sickest patients. Additionally, a standardized, evidence-based tool fosters consistency in practice across different levels of the health system, guiding less experienced clinicians in community hospitals and aiding triage in tertiary centers. It may also serve as an educational tool for trainees, reinforcing key predictors of severe dengue.

Although age < 55 years was identified as one of the predictive variables, the wide confidence interval (0.97–38.87) and borderline *p*-value (0.054) indicate a degree of statistical uncertainty. This may be due to the limited number of severe dengue cases relative to the overall sample size and variability in the age distribution within the study population. Therefore, this factor should be interpreted with caution. Future studies with larger and more balanced age groups are warranted to validate the predictive role of age in severe dengue progression.

Comparison with Existing Predictive Models

The findings of this study align with and extend previous research by offering a tailored, locally developed predictive tool. Several attempts have been made globally to develop practical scoring systems for dengue severity, each with its own strengths and limitations.

In a study conducted in Taiwan, logistic regression predictors were transformed into a simple numerical score to create a risk assessment tool for adults. While their model demonstrated moderate predictive ability (AUROC 0.848), it relied on parameters that may vary in prevalence across different populations, such as minor gastrointestinal bleeding [8]. Our model achieves a higher discriminatory performance (AUROC 0.88), suggesting superior predictive power in the Thai context. Similarly, a bedside score in New Caledonia, which also used similar clinical and laboratory parameters, reported a median AUROC of 0.88 in males and 0.80 in females [9]. The performance of our single-model score aligns with the higher end of this range, demonstrating robust predictive ability without the need for gender-specific stratification.

Beyond these examples, other models have shown utility in their respective populations. While models like the SMS Dengue Severity Score from India offer a simplified approach for adult populations, they rely on specific parameters that may be less accessible in routine practice. For instance, their score incorporates complex variables such as older age, which may have a different threshold in a Thai population, and prothrombin time or organ involvement, which require more specialized assessment and may not be routinely available in all settings. This contrasts with our model’s focus on readily accessible clinical signs and standard laboratory tests, making our tool more practical for use in resource-limited environments [10].

The Indonesian Dengue Score was designed to predict specific complications such as pleural effusion and/or ascites in adults. Although their model achieved excellent discrimination (AUROC = 0.88), its focus on specific complications and a different patient demographic limits its direct comparison with our comprehensive model for overall severe dengue in adults [11].

A study from a single center in Thailand developed a dengue risk score for adults with an excellent predictive ability (AUROC 0.902). However, it incorporated more specialized variables such as serum albumin, aspartate aminotransferase (AST), alanine aminotransferase (ALT), partial thromboplastin time (PTT), and anti-dengue IgM antibodies [16]. These parameters, while clinically useful, may not be routinely available in all settings. Furthermore, their derived score has a broad range from 0 to 38.6, which might be less straightforward for bedside calculation compared to our model. This contrasts with our model’s focus on readily accessible clinical signs and standard laboratory tests. Our score was developed using a multicenter Thai data set, ensuring its greater relevance for local application.

Compared with these existing models, our score holds a distinct advantage. While other systems may be effective in their specific settings, their direct applicability to Thailand is constrained by differences in patient demographics, serotype distribution, and clinical practice [8]. Furthermore, our model’s reliance on routinely available parameters makes it more practical for implementation in resource-limited settings than those requiring advanced diagnostics or complex calculations [9,10,11]. Our score was developed using a multicenter Thai data set, ensuring its relevance for local application. This approach aligns with and extends previous research in Thailand, as a comparable dengue risk score for Thai adults has also been developed, demonstrating the local relevance and effectiveness of this approach [22]. This localized development and reliance on readily accessible variables are key strengths that address a critical gap in the clinical management of adult dengue in Thailand.

In addition to model performance, it is also essential to consider the clinical reasoning behind the selection of individual predictors included in the scoring system. Among these, the role of age warrants further discussion given its inclusion as one of the explanatory variables.

The inclusion of age as one of the explanatory variables in the final model was based on both clinical rationale and supporting evidence from previous research. Although age < 55 years showed only a borderline statistical association in this dataset, several studies have consistently demonstrated that older age is associated with more severe disease manifestations, impaired immune response, and higher mortality risk in dengue infection. Given its biological plausibility and its role as a fundamental demographic factor that is easily obtainable at the point of care, age was retained in the model to ensure that clinically meaningful predictors were incorporated. This decision emphasizes the importance of integrating clinical relevance alongside statistical significance, thereby enhancing the interpretability and generalizability of the scoring system in real-world clinical settings.

This rationale-based inclusion strategy reflects one of the key strengths of the present study—its emphasis on clinical interpretability and real-world applicability—while acknowledging that future external validation remains necessary to confirm the model’s stability over time and across populations.

The moderate-risk group represented a substantial proportion of the study population and demonstrated intermediate clinical characteristics between mild and severe dengue cases. Patients in this category often exhibited warning signs, such as mild plasma leakage or moderate thrombocytopenia, but without organ dysfunction or shock. This finding suggests that the moderate group may represent a transitional phase in the disease spectrum, where timely recognition and appropriate clinical monitoring could prevent progression to severe outcomes. The relatively broad distribution of this group also reflects the heterogeneity of dengue manifestations among adults, underscoring the need for careful clinical assessment even in patients who do not initially meet the criteria for severe disease. Further studies exploring specific biomarkers or dynamic changes in laboratory parameters could help refine risk stratification within this intermediate category.

Limitations

Despite these strengths, several limitations should be noted. First, internal or external validation of the scoring system was not performed in this study due to the limited sample size and retrospective study design. Consequently, the model’s generalizability and predictive performance in different clinical settings or among adult populations with distinct characteristics—such as elderly patients or those with significant comorbidities—remain to be confirmed. Second, the retrospective design introduces the risk of information bias and incomplete data capture, as medical records from multiple hospitals vary in quality. Third, the study population was restricted to hospitalized adults, excluding mild cases managed in outpatient settings; this may limit generalizability. Fourth, the scoring system was developed based on predictors identified from a previous regression model, which may introduce potential bias or overfitting. Therefore, further validation using independent datasets is required to confirm the model’s robustness and generalizability. Fifth, the current study is that potential interactions among explanatory variables were not assessed. Investigating these interactions in future studies could provide additional insight into the relationships between predictors and severe dengue outcomes, potentially improving model performance while maintaining practical applicability. Six, the broad clinical range within the “moderate” category, which may have encompassed heterogeneous patient characteristics. This categorization was based on the original clinical criteria to maintain adequate case numbers for analysis. However, further subdivision of this group or the inclusion of additional clinical or laboratory parameters could improve discrimination between disease severities. Finally, potentially informative biomarkers (e.g., viral load, cytokines) were unavailable and therefore not included in the model.

Although this scoring system was developed using data from Thai patients, its potential applicability to other Southeast Asian populations with similar dengue epidemiology warrants further investigation. Validation in independent cohorts from neighboring countries will be essential to establish its generalizability and clinical utility across broader regional settings. While the model demonstrated good internal validity, external validation in diverse populations remains necessary before its routine clinical implementation. Importantly, although the scoring system shows promise for supporting clinical decision-making, it has not yet been implemented in routine practice, and formal feedback from clinicians has not been collected. Future studies are therefore warranted to assess its real-world usability, acceptability, and impact on patient management in clinical settings, in addition to confirming its predictive performance across broader populations.

Future Research

Future research should focus on addressing these limitations. External validation in diverse clinical settings is essential to confirm the reliability and generalizability of the score. Validation in elderly populations or adults with significant comorbidities would further broaden its clinical applicability. Integrating the score into electronic medical records or mobile health platforms could facilitate real-time use and data collection for continuous refinement. Hybrid models that combine this score with novel biomarkers may also enhance its predictive performance, particularly in referral hospitals. Additionally, a cost-effectiveness analysis should be undertaken to evaluate whether implementing the score reduces healthcare expenditure by streamlining admissions and optimizing resource allocation.

## 5. Conclusions

This study provides a simple, evidence-based scoring system that reliably predicts severe dengue using readily available clinical and laboratory data. The derived dengue infection severity score classified patients into low, moderate, and high risk, with outstanding discrimination of non-severe from severe groups. Its implementation in Thai hospitals could improve early risk stratification, guide appropriate levels of care, and strengthen healthcare system preparedness during dengue epidemics. With further validation, the score has the potential to become an integral component of clinical practice and public health strategies for dengue management.

## Figures and Tables

**Table 1:** Baseline demographic, clinical, and laboratory characteristics of patients categorized by dengue severity.

Baseline Characteristics	Severe	Non-Severe	*p*-Value
	n (%)	n (%)	
Demographic			
Female	54 (50.5)	260 (45.06)	0.342
Age (years), mean (SD)	27.1 (±10.8)	28.9 (±13.1)	0.125
BMI (kg/m^2^)	22.4 (4.9)	23.3 (5.1)	0.079
Underlying disease	5 (4.7)	45 (7.8)	0.315
Hypertension	2 (1.9)	28 (4.9)	0.206
Diabetes Mellitus	3 (2.8)	19 (3.3)	1.000
Dyslipidemia	1 (0.9)	16 (2.8)	0.495
Asthma	1 (0.9)	7 (1.2)	1.000
Clinical presentation			
fever	107 (100)	577 (100)	-
Headache	60 (56.1)	384 (66.6)	0.047
Myalgia	70 (77.0)	121 (74.7)	0.163
Retro-orbital pain	15 (14.0)	77 (13.3)	0.877
Bone pain	4 (3.7)	8 (1.4)	0.102
Joint pain	5 (4.7)	40 (6.9)	0.524
Abdominal pain	34 (31.8)	110 (19.1)	0.139
Vomiting	56 (52.3)	255 (44.2)	0.323
Cough	21 (19.6)	141 (24.4)	0.139
Diarrhea	22 (20.6)	119 (20.6)	1.000
Petechiae	19 (17.8)	87 (15.1)	0.469
Rash	4 (3.7)	44 (7.6)	0.214
Epistaxis	5 (4.7)	25 (4.3)	0.800
Bleeding from the gums	10 (9.4)	54 (9.4)	1.000
Severe bleeding *	50 (46.7)	19 (3.3)	<0.001
Hepatomegaly	3 (2.8)	6 (1.0)	0.154
Pleural effusion	11 (10.3)	7 (1.2)	<0.001
Rapid, weak pulse	40 (37.4)	0	<0.001
Hemodynamic, mean (SD)			
Systolic blood pressure (mmHg)	89.6 (17.8)	114.7 (11.9)	<0.001
Diastolic blood pressure (mmHg)	56.4 (14.4)	72.0 (10.0)	<0.001
Pulse pressure	33.2 (9.6)	42.8 (10.3)	<0.001
Hematological			
Hematocrit (%), mean (SD)	42.2 (5.3)	42.2 (7.6)	0.880
White blood cell (/µL), median (IQR)	3790 (2600, 5600)	3500 (2600, 5000)	0.569
Platelet (/µL), median (IQR)	48 (20, 73)	103 (71, 151)	<0.001
Neutrophils (%), mean (SD)	59.4 (19.7)	61.2 (16.9)	0.334
Lymphocyte (%), median (IQR)	25.7 (16, 36.3)	25 (17, 37)	0.817
Biochemical			
AST (U/L), median (IQR)	122 (67.5, 385)	83 (44, 133)	<0.001
ALT (U/L), median (IQR)	68 (34.5, 211.5)	45 (26, 89)	<0.001
Duration of admission (days), median (IQR)	4 (3, 5)	3 (2, 4)	0.001
In hospital dead	6 (5.6)	0	<0.001

Note: * Bleeding includes gastrointestinal bleeding, hematemesis, melena, menorrhagia, and hematuria. Abbreviations: IQR, interquartile range; AST, aspartate aminotransferase; ALT, alanine aminotransferase.

**Table 2:** Prognostic indicators of severe dengue infection in adult patients.

Prognostic Indicators	Multivariable OR * (95% CI)	*p*-Value
Age < 55 years	6.13 (0.97, 38.87)	0.054
Severe bleeding	20.75 (10.66, 40.38)	<0.001
Pleural effusion	10.23 (4.65, 22.54)	<0.001
Platelet ≤ 100,000 (/µL)	3.62 (1.10, 11.94)	0.035

Notes: * Odds ratio from multivariable logistic regression.

**Table 3:** Significant predictors of dengue infection severity and assigned item scores.

Predictors	Category	OR	95% CI	*p*-Value	Coefficient *	Score
Age < 55 years	yes	6.13	0.97, 38.87	0.054	1.81	1.5
	no	1.00	reference			0
Bleeding episodes	yes	20.75	10.66, 40.38	<0.001	3.03	2.5
	no	1.00	reference			0
Pleural effusion	yes	10.23	4.65, 22.54	<0.001	2.33	2
	no	1.00	reference			0
Platelet (/µL)	≤100,000	3.62	1.10, 11.94	0.035	1.29	1
	>100,000		reference			0

Notes: * Coefficients from multivariable logistic regression; OR, odds ratio; CI, confidence interval.

**Table 4:** Distribution of severe and non-severe groups into low, moderate, and high probability categories, likelihood ratio of positive (LHR+), and 95% confidence interval.

Probability Categories	Score	Severe (n = 107)		Non-Severe (n = 577)		LHR+	95% CI	*p*-Value
		n	%	n	%			
Low	≤2.0	7	6.5	302	52.3	0.12	0.06, 0.26	<0.001
Moderate	2.5–5.0	52	48.6	266	46.1	1.05	0.85, 1.30	0.674
High	≥5.5	48	44.9	9	1.6	28.76	14.55, 56.85	<0.001
Mean (SD)	-	4.6	(1.5)	2.5	(1.2)	-	-	<0.001

**Table 5:** Performance metrics of the dengue severity risk score at different cut-off levels.

Score Cut-Off	Classification	Sensitivity (%)	Specificity (%)	PPV (%)	NPV (%)	LHR^+^	LHR^−^
≥2.5	Low vs. Moderate/High	93.5 (100/107)	52.3 (302/577)	26.7 (100/375)	97.7 (302/309)	1.96	0.13
≥5.5	Moderate vs. High	44.9 (48/107)	98.4 (568/577)	84.2 (48/57)	90.6 (568/627)	28.76	0.56

Notes: PPV, Positive Predictive Value; NPV, Negative Predictive Value; LHR^+^, Positive Likelihood Ratio; LHR^−^, Negative Likelihood Ratio.

## Data Availability

The original contributions presented in this study are included in the article. Further inquiries can be directed to the corresponding author.
